# Self-Medication: potential risks and hazards among pregnant women in Uyo, Nigeria

**Published:** 2012-09-19

**Authors:** Festus Abasiubong, Emem Abasi Bassey, John Akpan Udobang, Oluyinka Samuel Akinbami, Sunday Bassey Udoh, Alphonsus Udo Idung

**Affiliations:** 1 Department of Psychiatry, Faculty of Clinical Sciences, College of Health Sciences, University of Uyo, Akwa Ibom State, Nigeria; 2 Department of Obstetrics & Gynaecology, Faculty of Clinical Sciences, College of Health Sciences, University of Uyo, Akwa Ibom State, Nigeria; 3 Department of Pharmacology, Faculty of Clinical Sciences, College of Health Sciences, University of Uyo, Akwa Ibom State, Nigeria; 4 Department of Family Medicine, Faculty of Clinical Sciences, College of Health Sciences, University of Uyo, Akwa Ibom State, Nigeria

**Keywords:** Self-medication, hazards, pregnant women, Nigeria

## Abstract

**Introduction:**

There is increasing evidence that self-medications among pregnant women are common in many developing countries. Despite the adverse impact on pregnancy, there are few programs available for their control. The objective of this study was to assess the level of self-medication amongst Nigerian pregnant women in order to determine possible harmful effects on fetus.

**Methods:**

Five hundred and eighteen 518 pregnant women, aged between 18 and 40 years, drawn from three General hospitals in Akwa Ibom State were assessed for self-medication and substance abuse using an instrument, adapted from a modified form of 117-item self-report questionnaire based on the WHO guidelines for students’ substance use survey.

**Results:**

Of the 518 pregnant women assessed, 375 (72.4%) indulged in one form of self-medication or the other; 143 (27.6%) used only drugs prescribed from the antenatal clinic. A total of 157 (41.9%) pregnant women self-medicate fever/pain relievers; 47 (9.1%) mixture of herbs and other drugs; 15 (4.0%) sedatives; 13 (3.5%) alcohol; while 5 (1.3%) used kolanuts. Reasons for using these substances range from protection from witches and witchcrafts, preventing pregnancy from coming out, for blood; poor sleep, fever and vomiting and infections. There was a significant difference in the rate of using analgesics (X2=9.43, p=0.001); and antibiotic (X2=4.43, p=0.001) among pregnant women who were highly educated compared to those with little or no education. However, the level of education has no impact in the usage of native herbs.

**Conclusion:**

This study shows that self-medication is common among pregnant women in our environment. There is need for adequate education of pregnant women during antenatal clinics on the potential danger of self-medication so as to prevent child and maternal morbidity and mortality.

## Introduction

Self-medication is common all over the world [1, 2]. In the face of current global economic downturn, a large number of countries are facing serious health challenges, with people finding it difficult to meet their health needs. In developed countries, self-medication is not uncommon, but the practice is guided because people are enlightened and could derive adequate information from various sources [3]. Consequently, it is often regarded as consumers’ luxury and very attractive. Evidence suggests that many people involved in self-medication tend to acquire knowledge of the practice from relatives, neighbours, medicine dealers, and sometimes media [4]. The situation in developing countries is frightening, where there is poor medical services and lack of professional control of pharmaceutical products [1]. This therefore forces people to self-medicate and various forms of substances and herbs are often used for different medical complaints [5, 6]. Even though the practice is high, there is scarcity of data on the impact on the people [7, 8]. A few of the studies that had been carried out have shown potential harmful effects on both the fetus and mothers usually exposed to unprescribed modern medications or traditional herbs [9]. Several factors including poverty, cultural perception of certain diseases’ entity and their perceived responses to indigenous medications have been widely reported as indicators in developing countries making the practice a necessity [4, 10, 11]. This makes it more dangerous as basic knowledge concerning the pharmacological properties of these substances/ drugs may be lacking.

One major consequence of self-medication that has not been properly given serious attention is substance abuse [12]. This could be a problem especially in rural communities, where native concoctions mixed with alcohol in the form of palmwine and locally-made gin are consumed on regular basis [9]. Although efforts aimed at reducing substance abuse and its associated health hazard are intensified in many countries, it is still a major problem especially in developing countries because of its adverse socioeconomic and health impact. In African countries, little is known about the potential effects of these substances on pregnancy. Many of them are readily available in many homes and are commonly used in the preparation of herbs and other concoctions. Furthermore, being local substances of traditional importance, their use is unrestricted even during pregnancy. There is increasing evidence that unborn babies exposed early to these substances may suffer from overwhelming morbidity and mortality [13].

In Nigeria, official corruption and increasing political instability have resulted in defective functional health institutions [14]. Although successive governments in the past have promised free healthcare for pregnant women, several obstacles including lack of sincerity in planning and implementation on the part of policy makers have made the gesture an impossible task. In addition, the attitude and low morale of staff in public healthcare clinics, as well as the cultural beliefs and perception have contributed immensely to alternative healthcare practice. Therefore, self-medication has become a norm and is widely practiced and patronized [7]. Though largely empirical and observational, evidence suggests that the practice may be high in rural areas with poor terrain, limited health facilities, high illiteracy level and poverty. Some of the substances used are locally formulated-mixtures with ingredients capable of resulting in abuse with serious health hazard on both the mother and fetus. However, no information is currently available on the impact of this practice on pregnancies and unborn child in our environment. This study therefore was aimed at examining the extent, nature and possible factors identified as being responsible for self-medication among pregnant women in Uyo, a community in Niger Delta region, Nigeria.

## Methods

**Location of the study:** This study was carried out at three General Hospitals in Akwa Ibom State. The State is situated in the South-South geopolitical zone of Nigeria and is one of the oil producing States in Niger Delta region of Nigeria. It has a population of 3.9 million people. Altogether the state consists of 31 local government areas with one general hospital in each local government.

**Data Collection:** Using a two-stage random sampling method, 518 pregnant women were drawn for the study. The first stage of the sampling by balloting selected 3 General Hospitals as locations of the study. Then using a systematic random sampling method with a sampling interval of three, five hundred and eighteen pregnant women were randomly selected and screened for self medication and substance abuse, using a questionnaire adapted from a modified form of 117-item self-report instrument based on the World Health organization guidelines for students’ substance-use surveys [15]. Commonly available antibiotics, analgesics, antimalaria, haematenics antihelmentics and traditional medicine/herbs were added to the list of substances in the questionnaire. Participants were also asked indicate to the reasons for using the substances. Information on age, marital status, educational level and occupation were elicited through a semi-structured sociodemographic questionnaire. Every 3rd pregnant woman who presented in the clinic for antenatal care on each day during the period of the study was recruited and assessed. The interval of 3 derived by dividing the target population which was 1,430 women by the sample size (410), calculated from the formula: N=z2pq/d. A purposive sample size of 518 subjects was eventually assessed. Permission to carry out the study was obtained from the State Hospital management Board through a letter from the University of Uyo Teaching Hospital.

**Data analysis:** The results of the study were analyzed using Statistical Package for Social Sciences (SPSS 17.0). The proportion of the pregnant women involved in the use of different substances was found from the respondents. Sample means and percentages were calculated from which simple frequency tables were created. Standard deviation from the mean was calculated and comparisons of categorical data were done using Chi-square. The P-value of less than or equal to 0.05 was used to determine the statistical significance.

## Results

Table 1 shows the sociodemographic characteristics of pregnant women. Of the 518 pregnant women assessed, 45 (8.7%) were aged below 20 years, 231 (44.6%) between 20 and 29 years, 214 (41.3%) 30-39 years, while 28 (5.4%) were aged above 40 years. A total of 51 (9.9%) had no formal education, 101 (19.5%) primary education, 227 (43.8%) secondary and 139 (26.8%) post secondary education; 29 (5.6%) of the women were single, 421 (81.3%) married, 64 (12.4%) were living together without marriage, while 11 (2.1%) were separated, divorced or widowed. Two hundred and eighty two (54.4%) women were in business, 110 (21.2%) were civil servants, 54 (10.4%) farmers, while 72 (13.9%) were full-time house wives.

**Table 1:** Socio-demographic characteristics of the pregnant women

Variables	Frequency	Percentage
**Age (years)**		
<20	45	8.7
20-29	231	44.6
30-39	214	41.3
>40	28	5.4
**Educational Level**		
No formal education	51	9.9
Primary school	101	19.5
Secondary school	227	43.8
Post-secondary school	139	26.8
**Marital Status**		
Never married	29	5.6
Married	414	79.9
Live together w/out married	64	12.4
Sep/divorced/widow	11	2.1
**Occupation**		
Full-time h/wife	72	14.0
Farming	54	10.4
Business	282	54.4
Civil servant	110	21.2

One hundred and forty three (27.6%) out of the 518 pregnant women used drugs prescribed only from the antenatal clinics throughout the pregnancy. Table 2 shows the prevalence of self-medication amongst pregnant women. A total of 375 (72.4%) pregnant women indulged in one form self-medication or the other for various reasons. Of this, 138 (26.6%) abused or used antibiotics without prescription, 157 (30.3%) fever/pain relievers, 47 (9.1%) used alcohol related-mixture of herbs and other drugs, 15 (4.0%) sleeping medicines (valium, lexotan, librium), while 13 (2.5%) used alcohol in the form of palmwine, brewed beer (Guinness stout beer) and 5 (1.0%) used kolanuts. Seventy one (51.4%) out of 138 women that abused antibiotics; 105 (66.9% out of 157 that used fever/pain relievers; 33 (70.2%) out 47 that used alcohol-related mixtures and 11 (73.3%) out of 15 that used sleeping medicines, had post-secondary education, while 33 (70.2%) out 47 pregnant women that used alcohol-mixtures were mostly without formal education and those with primary education. More than 70% of women with little or no education found it difficult to assess health facilities and reasons for using herbs were either for blood, protection from witches/witchcrafts, or for preventing pregnancy from coming out.

**Table 2:** Prevalence of self-medication and use of substances amongst the respondents.

Substances	Frequency n (%)
Kolanuts	5 (1.0)
Coffee	-
Tobacco/Cigarette	-
Sedatives (Lexotan, Valium, Phenobarb)	15 (2.9)
Analgesics (Pain relievers or killers)	157 (30.3)
Antibiotics (Septrin,Tetracycline, Ampicllin)	138 (26.)
Mixture native herbs and other drugs	47 (9.1)
Alcohol (P/wine, local liquor, beer, gin)	13 (2.5)
Indian Hemp	-
Cocaine	-
Heroin	-
Anabolic steroids	-
Lysergic diethylamide Acid (LSD)	-

Table 3 shows the relationships between self-medicated substances and the levels of education of the pregnant women. Pregnant women were divided into low and high educational groups. The low level of education were conceived by the researchers to include those with primary and those with no formal education, while high level of education was made up of women from secondary school education level and above. Antibiotics, analgesics and alcohol in the form of palmwine and brewed beer (Guinness stout beer) were mostly abused by educated pregnant women. A total of 131 (25.3%) pregnant women with high educational level, compare with 26 (5.0%) with low level used fever/pain relievers during current pregnancy (X^2^=9.43, p=0.001). This was statistically significant. Also, 93 (17.9%) pregnant women with high against 45 (8.7%) with low level of education used antibiotics mostly ampicillin, tetracycline, chloramphenicol and septrin (X^2^=4.43, p=0.001); 10 (1.9%) with high against 5 (1.0%) with low level used sedatives such as valium, librium and lexotan. A total of 26 (5.0%) pregnant women with low education and 21 (4.1%) with high education used mixture of herbs and other drugs (X^2^=0.58, p=0.559), while 8 (1.5%) of pregnant women with low education as against 5 (1.0%) with high education used alcohol (X^2^=0.56, p=0.552).

**Table 3:** Relationship between self-medicated substances and levels of education of the pregnant women

Substances	Education			
	Low	High		
	n (%)	n (%)	X^2^	P-value
Kolanuts	3 (0.6)	2	0.06	0.955
Sedatives	5 (1.0)	10	1.11	0.268
Analgesics	26 (5.0)	131	9.34	0.001*
Antibiotics	45 (8.7)	93	4.43	0.001*
Mixture of N/herb	26 (5.0)	21	0.58	0.559
Alcohol	8 (1.5)	5	0.56	0.572

* Statistically significant

## Discussion

The findings from this study suggest that the practice of self-medication is common among pregnant women in our environment. In this study, various substances ranging from traditional preparations/substances including kolanuts and local gin to orthodox medications were found to have been used by the pregnant women. Although the types of herbs vary according to place, different culture and custom, this is similar to reports in earlier studies within and outside Nigeria [2, 5–8, 13]. These findings are frightening and very serious in view of the poor knowledge of the safety profiles of these substances among the women [11]. Though the rates of use of some of the substances in this study are low, the difficulty in estimating the right and adequate dosages may pose serious problem [1]. One major concern would be the potential harmful effects on the fetus. Therefore, the use of substances such as alcohol and alcohol-related mixtures for whatsoever reason during pregnancy should be discouraged. Evidence suggests that substances like alcohol is an important risk factor for burden of disease and social harm, accounting for 3.2% of all deaths and 4.0% of all disability adjusted life years (DALYs) globally [16]. There is also increasing evidence that exposing fetus to alcohol in utero may result in intrauterine growth and postnatal development retardation [17]. If the exposure is in the early month of pregnancy, these children could suffer from serious complications including, congenital malformation, mental retardation or even intrauterine fetal death [11]. This therefore highlights the need for effective programmes that would target on prevention of self-medication among pregnant women and intervention to arrest complications.

Our study shows variation in the practice and use of self medication among the participants. In this study, about 25% and 35% of the pregnant women with high level of education used analgesics and antibiotics respectively. This, when compared with 6.9% and 12.0% of them with low level of education involved in the use of the same substances, is very significant. Also about 6.9% of women with low education self-medicate herbs compared to 5.6% of those with high education. These findings seem to suggest that the level of education influences the type and nature of substances used. Although earlier studies have association self-medication with factors such as self-employment, unemployment and third trimester of pregnancy, the use of herbal medicines has been strongly linked with low education [18]. However, several reasons may be responsible for this differential practice in this study. First, the use of analgesics, antibiotics and alcohol by women with high level of education and traditional herbs by those with low level of education may be due to health beliefs, which to a large extent, determines the emotional and behavioural responses to illness [19]. These beliefs involve expectancy and perceived benefits, as well as outcome for initiating and maintaining treatment. There is abundance of evidence that this perception plays a significant role in health-seeking behaviour and pathway to care [10]. Secondly, it is possible that inspite of the poor knowledge of the efficacy of some of these substances among pregnant women with low education, alternative care is widely known to be readily accessible and affordable [18]. Finally, high education may have increased awareness, more opportunities and options for different levels of care. In addition, the conscious effort for quick outcome may also have explained the high patronage of traditional herbs by the pregnant women with high level of education in this study.

The findings of this study seem to highlight a more disturbing trend of practice among pregnant women in our environment. An inference from the findings suggests that there is increasing trend of dual or simultaneous consultation during pregnancy. This is evident by the various agents seen to have been taken by the pregnant women, mostly traditional mixtures and herbs. This cuts across both educated and non-educated women. A possible explanation may be the belief on the efficacy of these substances on indigenous/traditional illnesses [5, 14]. Although various reasons have been proffered for this practice, the implications could be overwhelming, as issues related to possible drug interactions could be a major problem to both mother and the unborn child [13]. Therefore, health care providers must be aware of this trend, in order to weigh the therapeutic benefits to mothers and the potential risk to the developing fetus [9]. It is essential to routinely inquire about the woman′s self-medication practice so as to provide appropriate advice during antenatal care. Studies have shown that some of these preparations are often prepared under unhygienic conditions and has the possibility of causing microbial infection [5]. In an environment such as ours, where every illness is attributable to supernatural deities and magical powers, judging from our cultural perception on causation of certain illnesses, this could portend serious danger to care of individuals with problems [20–22]. Also in this study, various orthodox medical regimens were found to be used by pregnant women. A significant proportion, 42% of pregnant women self-medicate analgesics, 37% antibiotics and 4% various forms of sedatives. Even though, these are modern medicine, the act of self-medication could be dangerous, considering the possible poor knowledge of pharmacodynamics of these medicines [4]. This is more worrisome as the sources and instructions concerning the use of these medicines are not from professionals. One major concern would be issue of inadequate dosing and resistance with respect to antibiotics. The widespread use of suboptimal dosages of antibiotics is a major threat, contributing to the development of resistant bacterial strains [23]. Antibiotics, such as septrin, choramphenicol and tetracycline are dangerous and contraindicated during pregnancy [24–27]. Apart from the resistance that may result from inadequate dosing, exposing fetus to them in early months of pregnancy may result in various complications, such as, kernicterus, gray baby syndrome and respiratory distress.

One other serious danger in our environment is the issue of counterfeit and expired drugs. This is a major threat to curative medical care, especially in rural communities [28]. The condition is made worse by the fact that the control and sale of almost all drugs are lacking. Therefore, there is availability and increased use of these counterfeits and expired drugs among the people, and pregnant women are not spared. This is very rampant in many rural settings, and with the twin problems of poverty and poor level of education, this could be a source of many unresolved medical conditions during pregnancy. The implication would be the potential harmful effect on the fetus. Therefore, there is need to put in place measures to either control or ban the sale of drugs by unauthorized persons other than professionals. Efforts must also be made to make sure that drugs/medicines are only available to individuals on prescription from a physician following a consultation.

Interestingly, the findings of this study also revealed that pregnant women in our environment do not indulge in a more potent-habit forming substances. This is clearly demonstrated in this study as it has shown that no pregnant woman smoked cigarette/tobacco or used any other potent substances, such Indian hemp cocaine or heroin. Several reasons may have contributed to this. First, these substances except tobacco, cigarette and Indian Hemp to a large extent are foreign to the environment and the knowledge of their usefulness, if any is limited. Although it is important to emphasize that the non use of these substances does not mean that they are not available. Secondly, the cost of obtaining them may be very high and affordability may also influence their usage. Finally, the custom, which restricts women from using certain substances, even in times of ill-health, may have also contributed to their non use. The major constraints in this study are the difficulty in determining the outcome of these substances on both the mothers and fetus. Also being a self-report, the disclosure of information concerning the practice of self-medication may be biased. This study involves only the pregnant women who indulged in self-medication, not necessarily because of sickness, but due to physiological changes associated with pregnancy. Therefore, the perceived benefits leading to their continued use may be exaggerated. There is also the problem of itemizing and translating the various types of herbs into a common language that may easily be understood. This is an isolated hospital-based study; therefore, the results cannot be generalized. Finally, an instrument adapted from a modified form of 117-item self-report questionnaire based on the World Health organization guidelines for students’ substance-use surveys was used in the assessment; therefore introducing substances like ‘our traditional herbs’ not originally captured in the questionnaire can affect its validity and reliability.

## Conclusion

The findings of this study have shown that the practice of self-medication among pregnant women is on the increase in our environment. Therefore, there is need to reduce this practice by improving the quality of antenatal care services to include adequate health education on major issues capable of influencing the health behaviours of our pregnant women. In order to achieve the millennium development goal aimed at reducing child and maternal mortality, there is need to upgrade healthcare facilities and embark on regular massive enlightenment campaigns, especially in rural areas to encourage increased healthcare services utilization. Since many of these pregnant women for reasons known to them and irrespective of their level of education, also patronize churches and traditional birth attendance homes for delivery, there is need for education of the public on the danger of self-medication and the potential harmful effect on the unborn child. This will ensure attitudinal change and encourage safer practice. More importantly, there is a pressing need for strict drug prescription control and monitoring to ensure that drugs are not prescribed and sold indiscriminately by those without basic pharmacological knowledge.
